# Does the availability of a South Asian language in practices improve reports of doctor-patient communication from South Asian patients? Cross sectional analysis of a national patient survey in English general practices

**DOI:** 10.1186/s12875-015-0270-5

**Published:** 2015-05-06

**Authors:** Faraz Ahmed, Gary A Abel, Cathy E Lloyd, Jenni Burt, Martin Roland

**Affiliations:** Cambridge Centre for Health Services Research, Institute of Public Health, Forvie Site, University of Cambridge School of Clinical Medicine, Box 113, Cambridge Biomedical Campus, Cambridge, CB2 0SR UK; Faculty of Health & Social Care, The Open University, Walton Hall, Milton Keynes, MK7 6AA UK

**Keywords:** Doctor-patient communication, Ethnic minority, South Asians, Doctor–patient relationship, Ethnicity, Inequities

## Abstract

**Background:**

Ethnic minorities report poorer evaluations of primary health care compared to White British patients. Emerging evidence suggests that when a doctor and patient share ethnicity and/or language this is associated with more positive reports of patient experience. Whether this is true for adults in English general practices remains to be explored.

**Methods:**

We analysed data from the 2010/2011 English General Practice Patient Survey, which were linked to data from the NHS Choices website to identify languages which were available at the practice. Our analysis was restricted to single-handed practices and included 190,582 patients across 1,068 practices. Including only single-handed practices enabled us to attribute, more accurately, reported patient experience to the languages that were listed as being available. We also carried out sensitivity analyses in multi-doctor practices.

We created a composite score on a 0-100 scale from seven survey items assessing doctor-patient communication. Mixed-effect linear regression models were used to examine how differences in reported experience of doctor communication between patients of different self-reported ethnicities varied according to whether a South Asian language concordant with their ethnicity was available in their practice. Models were adjusted for patient characteristics and a random effect for practice.

**Results:**

Availability of a concordant language had the largest effect on communication ratings for Bangladeshis and the least for Indian respondents (p < 0.01). Bangladeshi, Pakistani and Indian respondents on average reported poorer communication than White British respondents [*-2.9* (95%CI -4.2, -1.6), *-1.9* (95%CI -2.6, -1.2) and *-1.9* (95%CI -2.5, -1.4), respectively]. However, in practices where a concordant language was offered, the experience reported by Pakistani patients was not substantially worse than that reported by White British patients (-0.2, 95%CI -1.5,+1.0), and in the case of Bangladeshi patients was potentially much better (+4.5, 95%CI -1.0,+10.1). This contrasts with a worse experience reported among Bangladeshi (*-3.3,* 95%CI -4.6, -2.0) and Pakistani (*-2.7,* 95%CI -3.6, -1.9) respondents when a concordant language was not offered.

**Conclusions:**

Substantial differences in reported patient experience exist between ethnic groups. Our results suggest that patient experience among Bangladeshis and Pakistanis is improved where the practice offers a language that is concordant with the patient’s ethnicity.

**Electronic supplementary material:**

The online version of this article (doi:10.1186/s12875-015-0270-5) contains supplementary material, which is available to authorized users.

## Background

Good communication is an essential skill and core feature of high quality general practice (GP) and patient-centred care world wide [1-3]. South Asians (i.e., Bangladeshi, Indian and Pakistani) are one of the largest ethnic minority groups in the UK [4], and results from the annual national GP Patient Survey in England show that they repeatedly reported poorer experience of GP compared to their White British counterparts [5,6]. The health of ethnic minorities is increasingly gaining interest and importance in the European policy arena, particularly as migrant and ethnic minority populations are both substantial and growing [7]. The 2011 census showed that 14 percent of the UK population reported their ethnic group as non-White; over half of these (7.5% of total UK population) reported their ethnicity as Asian/Asian British [4]. Approximately eight percent of residents living in England and Wales speak a main language other than English, and of those 20 percent (864,000) speak limited or no English at all [8]. Previous studies from Europe have identified language barriers as one of the main problems that undermine both the accessibility of health services and quality of care for migrants [7].

Not only are language barriers a concern for persons who come to England from overseas with pre-existing language difficulties, individuals may continue to experience language barriers even after settling for a number of years [7]. What is more, for both recent migrants and settled (e.g., settled second or third generation) ethnic minorities there still remains a range of other social and cultural factors contributing to ethnic variations in the experience of healthcare. These include unfamiliarity with rights/entitlements, lack of understanding or sensitivity towards minority cultures in health policy and practice, social exclusion, and direct or indirect discrimination [9-12]. Barriers to effective communication are the source of many of these problems, and there is evidence to suggest that barriers to communication are not entirely overcome by the use of interpreters [9,13]. Interpersonal barriers to communication, which may result from language and/or cultural differences between a patient and their doctor, may be reduced if the language spoken by a doctor and/or his or her ethnicity concords (i.e., matches) with the patients’ characteristics [14].

When a doctor and patient share the same language or ethnicity, this is termed language-concordance or ethnicity-concordance respectively. Concordance of language and ethnicity between doctors and their patients may improve reported patient satisfaction [15], communication and quality of interpersonal care [16,17], and reduced reported adverse medication effects and confusion with medication instructions [18]. Evidence of whether language or ethnicity concordance between doctors and their patients has a positive effect among ethnic minorities in English general practices is however limited [14]. This paper examines how one specific aspect of health care experience, doctor-patient communication, varies between South Asians and White British respondents when a South Asian language is available at a practice.

Our primary analysis was restricted to single-handed practices, in order to enable us to attribute reported patient experience more accurately to the languages that were reported to be available in practices. A single-handed practice in our study is defined as a practice where there is a general practitioner, who is not in partnership with another general practitioner [19]. A single-handed practice in the UK health system may from time to time have other staff, such as doctors in assistant, salaried or locum roles. However, by restricting the sample to single-handed practices, we greatly increase the chance that the experience reported by patients relates to doctors speaking the language advertised as being offered by the practice. Previous work from our research group has shown the South Asian report poorer experiences of doctor-patient communication in English general practices [20]. The aim of this study was to examine how patient reports of doctor-patient communication scores vary when a South Asian (i.e., Bangladeshi, Indian and Pakistani) patient is seen at a practice where a language (spoken by doctor only) was available that was concordant with the patient’s self-reported ethnicity.

## Methods

### Datasets

We included three datasets in the analysis:

1. **National GP Patient Survey (GPPS):** This survey asks about the experience of primary care patients in relation to their access to and experience of primary care. The 2010/2011 survey was sent to 5.6 million patients registered with 8,387 practices in England, with a response rate of 36% (1,994,410). This survey measures interpersonal aspects of care by looking at seven items of doctor-patient communication: i) provision of sufficient time; ii) asking about the patient’s symptoms; iii) listening to the patient; iv) explanation of tests and treatments; v) involving the patient in decisions about care; vi) treating the patient with care and concern; vii) taking patients’ problems seriously. It also includes other patient self-reported items, such as the patient’s ethnicity (16-categories as classified by the 2001 UK Office of National Statistics census [21]), gender, age, self-rated health status and presence of a longstanding psychological or emotional condition. In addition, a measure of socioeconomic status based on the postal code of the patient’s residential area was included in the dataset, coded as quintiles of deprivations for each patient [20,22,23]. Details of the survey and method of its administration have been published elsewhere [24]. Translated versions of the GP Patient Survey are available for Bangladeshi, Indian and Pakistani respondents in the appropriate languages (i.e., Bengali, Hindi and Urdu).
2. **2010 GP Census:** These data are collected by the Department of Health and record numbers and details of general practitioners in England. The census includes information on general practitioner’s practice staff, patients and the services they provide.
3. **NHS Choices language Dataset (2011/2012):** NHS Choices is a national website which provides information on health services and general practices in England for patients and the general public (http://www.nhs.uk/). From this dataset, we extracted the additional languages (i.e., a language other than English) which the practice advertises as being spoken by the doctor at practice, and not by any other staff members, as well as the details of their practice (name and address). This data is routinely updated by the local NHS Primary Care Trusts (now replaced by Clinical Commissioning Group). We coded all the languages (in addition to English) offered within each practice.

Additional file 1 presents the summary of the datasets used.

### Dataset linking

We linked the 2010/2011 National GP Patient Survey with the 2010 GP Census data (to identify single-handed practices) through a unique practice code. This combined dataset was then linked to the NHS Choices 2011-2012 language dataset by practice postcodes (16.9% (214) of the single-handed practices were not included as they shared the same postcodes), to classify which languages in addition to English were available at each practice.

### Analysis

**Figure 1:**
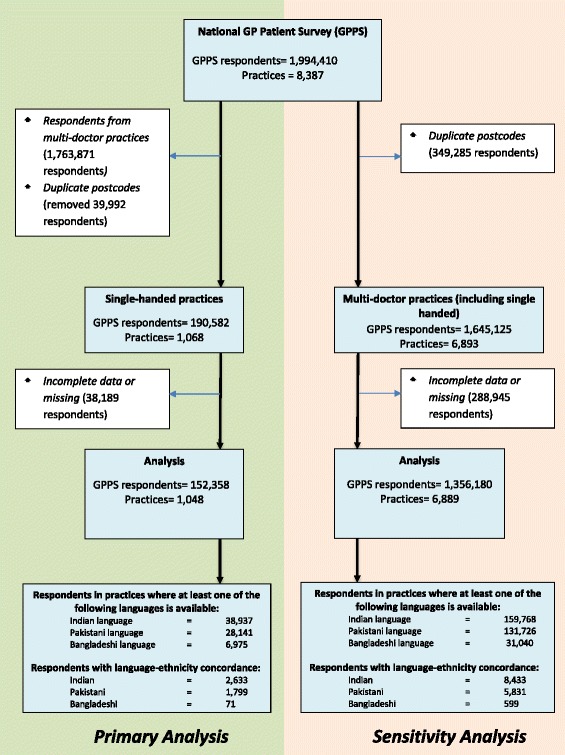
Selection of respondents in the analysis.

We examined the responses of 190,582 respondents across 16 ethnicity categories in 1,068 single handed practices. Of these, 38,224 respondents were excluded due to incomplete and missing data for the following variables; age, ethnicity, gender, self-rated health status, self-reported presence of a mental health condition and quintiles of deprivation and composite doctor-patient communication score (see Figure 1 for details). We used the five-point ordinal scales of the doctor-patient communication questions from the GP Patient Survey, and linearly rescaled to a 0-100 range (100 equating to the most favourable experience) [20,25-27]. The seven communication items of the GP Patient Survey have high reliability (Cronbach’s alpha = 0.99), which strongly suggests that they form a unidimensional scale [27]. A single composite 0-100 score was calculated as the mean of the seven uni-dimensional items for all respondents who answered at least four of the seven items [20,23,27].

Using NHS Choices data, we combined all the languages reported to be spoken by the doctors in a particular practice. Table 1 lists the South Asian languages (i.e., Bangladeshi, Indian and Pakistani) listed as available at general practices in England, and how we assigned them to a specific ethnic group. We created dichotomous variables for each of the South Asian ethnic groups for ‘language-ethnicity concordance’ (coded 1 if a South Asian respondent was seen at a practice where a concordant South Asian language was available; and 0 if a South Asian respondent was seen at a practice where there was no concordant language available).

**Table 1:** **NHS Choices language dataset for England**

**Ethnic group (Assigned language set)** ^Ɨ^	**Language (NHS Choices dataset)**	**Number of single and multi-doctor practices offering**	**% of all English practices offering** ^ƗƗ^	**Number of single-doctor practices offering**	**% of single-doctor practices offering** ^ƗƗƗ^
**Bangladeshi**		**229**	**2.73**	**31**	**2.96**
	Bengali	221	2.64	31	2.96
	Bangladeshi	8	0.10	0	0.00
**Pakistani**		**890**	**10.61**	**152**	**14.50**
	Urdu	752	8.97	135	12.88
	Punjabi or Panajabi^*^	613	7.31	96	9.16
	Mirpuri	18	0.21	5	0.48
	Sindhi	14	0.17	1	0.10
	Pushto or Pashto*	15	0.18	6	0.57
	Kashmiri	9	0.11	4	0.38
**Indian**		**1086**	**12.95**	**206**	**19.66**
	Hindi	884	10.54	174	16.60
	Punjabi or Panajabi^**^	454	5.41	73	6.97
	Gujarati	303	3.61	55	5.25
	Tamil	222	2.65	36	3.44
	Malayalam	52	0.62	9	0.86
	Marathi	46	0.55	7	0.67
	Kannada	44	0.52	8	0.76
	Telugu	44	0.52	6	0.57
	Assamese	8	0.10	1	0.10
	Oriya	4	0.05	1	0.10
**Total number of practices offering a South Asian language (i.e., Bangladeshi, Pakistani, Indian)**		**1,354**	**16.14**	**236**	**22.52**

^Ɨ^Languages were categorised by ethnic group after (a) reviewing literature on common languages spoken in the UK by South Asians, and (b) reviewing common languages spoken in the origin country (Bangladesh, Pakistan and India) using data from the World Factbook 2013-14 (https://www.cia.gov/library/publications/the-world-factbook/fields/2098.html).

^ƗƗ^Total practice in England = 8,387.

^ƗƗƗ^Total single-handed practices in our analysis = 1,048.

*This language was assigned to the Pakistani ethnic group, since majority of the doctors offering it also spoke another Pakistani language.

**Punjabi or Panajabi was also present alongside another Indian language.

We used mixed linear regression models that included patient socio-demographic variables (categorical age, ethnicity, gender, self-rated health status, self-reported presence of a mental health condition and quintiles of deprivation) as fixed effects and a random effect for practice.

We constructed two main models for single-handed practices:

1. An initial model not considering language-ethnicity concordance, which allowed us to estimate the difference in doctor-patient communication scores between White British and South Asian respondents in single-handed practices after adjusting for age, gender, health status, presence of a long-standing psychological or emotional condition, deprivation and practice. This model largely recreated previous GPPS analyses on the recent 2010/2011 dataset [20].
2. A second model including an additional effect for language-ethnicity concordance (i.e., patient’s self-reported ethnicity matching with the language available at a practice) for each of three South Asian ethnic groups (i.e., Bangladeshi, Indian and Pakistani). This allowed us to estimate the same differences (as model 1) in doctor-patient communication scores between White British and South Asian respondents separately where there is and is not a South Asian language-ethnicity concordance.

### Sensitivity analysis

We repeated the analyses including multi-doctor practices (where there was more than one doctor per practice – see Figure 1) to examine whether the effects of concordance still remained. We excluded 1,529 (18.2%) practices, as they shared the same postcodes.

We used Stata 11.2 for all analysis.

### Ethical approval

The GP Patient Survey is a service evaluation and GP Census is routinely collected NHS data, neither of which requires research ethics committee approval for their use. The data used from the NHS Choices website is in the public domain.

## Results

According to the NHS Choices data, at least 1,354 or 16% of the practices in England had a South Asian language available in their practice. Indian, Pakistani and Bangladeshi languages were reported as being available in 1,086 (13.0%), 890 (10.6%) and 229 (2.7%) of practices in England, respectively. In our analysis of single-handed practices Indian, Pakistani and Bangladeshi languages were reported as being available in 206 (19.7%), 152 (14.5%) and 31 (3.0%) practices, respectively.

Table 2 details the characteristics of survey respondents from single-handed practices. There are slightly more female respondents (55.2%) than male and the most common age group is 55 to 64. Just over 70% of respondents describe themselves as White British, with 5.1%, 2.7% and 0.7% of respondents describing themselves as Indian, Pakistani and Bangladeshi respectively.

**Table 2:** **Difference in reports of doctor-patient communication (scale 0-100) among survey respondents from single-handed practices (Model 1)** ^Ɨ^

		**Mean Score (0-100)**	**Survey respondents**		Score Difference*	**P-value**
**Variable category**			**(n)**	**(%)**	**Difference (SE)**	
**Gender**						**<0.0001**
	Male	90.4	83,446	44.8	**Reference**	
	Female	89.8	102,833	55.2	-0.6 (-0.8, -0.4)	
**Age**						**<0.0001**
	18 to 24	80.6	9,589	5.2	-9.9 (-10.4, -9.4)	
	25 to 34	81.3	22,429	12.1	-9.1 (-9.5, -8.7)	
	35 to 44	85.6	28,084	15.1	-4.8 (-5.1, -4.4)	
	45 to 54	88.1	32,391	17.4	-2.3 (-2.7, -2.0)	
	55 to 64	90.4	36,381	19.6	**Reference**	
	65 to 74	93.5	31,784	17.1	3.1 (2.7, 3.4)	
	75 to 84	94.5	19,533	10.5	4.1 (3.7, 4.5)	
	85+	93.7	5,624	3.0	3.3 (2.6, 4.0)	
**Ethnicity**						**<0.0001**
***White***	White British	90.4	131,570	70.4	**Reference**	
	Irish	91.0	3,024	1.6	0.6 (-0.2, 1.4)	
	Any other White background	87.0	10,989	5.9	-3.4 (-3.8, -2.9)	
***Mixed***	White and Black Caribbean	90.1	504	0.3	-0.3 (-2.3, 1.6)	
	White and Black African	91.6	427	0.2	1.2 (-1.0, 3.4)	
	White and Asian	87.8	473	0.3	-2.6 (-4.6, -0.6)	
	Any other Mixed background	87.7	723	0.4	-2.7 (-4.4, -1.0)	
***South Asian***	Indian	88.5	9,513	5.1	-1.9 (-2.5, -1.4)	
	Pakistani	88.5	4,991	2.7	-1.9 (-2.6, -1.2)	
	Bangladeshi	87.5	1,373	0.7	-2.9 (-4.2, -1.6)	
	Any other Asian background	89.2	4,703	2.5	-1.2 (-1.9, -0.6)	
***Black***	Black Caribbean	90.1	3,647	2.0	-0.4 (-1.2, 0.4)	
	Black African	91.1	4,989	2.7	0.6 (-0.1, 1.3)	
	Any other Black background	91.5	1,412	0.8	1.1 (-0.2, 2.3)	
***Chinese***	Chinese	85.3	1,152	0.6	-5.1 (-6.4, -3.9)	
***Other ethnic group***	Any other ethnic group	88.3	7,447	4.0	-2.1 (-2.7, -1.6)	
**Deprivation**						**0.4394**
	“1” (least deprived)	90.4	16,794	8.8	**Reference**	
	“2”	90.2	26,892	14.1	-0.3 (-0.7, 0.2)	
	“3”	90.0	36,565	19.2	-0.4 (-0.9, 0.1)	
	“4”	90.1	48,316	25.4	-0.3 (-0.8, 0.2)	
	“5” (most deprived)	90.3	62,015	32.5	-0.2 (-0.6, 0.3)	
**Self-reported health status**						**<0.0001**
	Excellent	90.4	15,425	8.4	**Reference**	
	Very good	86.2	49,775	27.0	-4.2 (-4.6, -3.8)	
	Good	82.4	65,486	35.5	-8.0 (-8.4, -7.6)	
	Fair	80.4	40,006	21.7	-10.0 (-10.5, -9.6)	
	Poor	79.6	13,910	7.5	-10.8 (-11.3, -10.3)	
**Long-standing psychological or emotional condition**						**0.0102**
	No	90.4	10,611	6.3	**Reference**	
	Yes	91.0	157,442	93.7	0.6 (0.1, 1.0)	

*Coefficients were also adjusted for a random effect for practice.

^Ɨ^Excluding the effects of a respondent being seen in a practice where a concordant language was available (model 1).

-Models carried out with Stata xtmixed procedure (fit model via maximum likelihood, ml), without robust standard errors.

### Main findings

**Figure 2:**
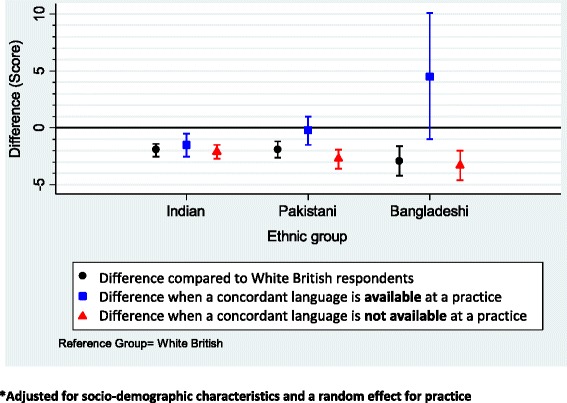
Effect of language/ethnicity concordance on mean doctor-patient communication score: South Asians compared to White British respondents.

Substantial differences in reported patient experience existed between ethnic groups (p < 0.0001) when compared to their White British counterparts (Table 2). There was strong evidence (p < 0.0004) to suggest that scores for doctor patient communication varied according to whether or not a concordant language was available at single-handed practices (based on a likelihood-ratio test comparing model 1 - without language-ethnicity concordance, and model 2 - with language-ethnicity concordance). There was also evidence (p = 0.0109) that the effect of language-ethnicity concordance varied within the South Asian group (i.e., Bangladeshi, Indian and Pakistani–based on a likelihood-ratio test comparing model 2 with a model where the language-ethnicity concordance effect was constrained to be constant across the three ethnic groups). This is summarised in Figure 2 and Table 3, where it can be seen that the availability of a concordant language had the largest effect for Bangladeshi respondents but little effect for Indian respondents.

**Table 3:** **Effect of concordance on the average doctor-patient communication difference for South Asians, when compared to White British respondents (single-handed practices)**

**Ethnic group**	**Model 1:**		**Model 2:**	
	**Mean Score (0-100)**	Average difference compared to White British respondents*	**Average difference compared to White British respondents when a concordant language is:**	
			Available at practice**	***Not available at practice*** ^**‡**^
Indian	88.5	**-1.9** (-2.5, -1.4)	**-1.5** (-2.5, -0.5)	**-2.1** (-2.7, -1.5)
Pakistani	88.5	**-1.9** (-2.6, -1.2)	**-0.2** (-1.5, 1.0)	**-2.7** (-3.6, -1.9)
Bangladeshi	87.5	**-2.9** (-4.2, -1.6)	**4.5** (-1.0, 10.1)	**-3.3** (-4.6, -2.0)
	**p < 0.0001** ^Ɨ^		**Likelihood-ratio test: p = 0.0109** ^ƗƗ^	

Both models were adjusted for age, gender, deprivation, self-rated health status, presence of a mental health condition, and a random effect for practice.

^**Ɨ**^Joint test of the differences of South Asians from White British.

^**ƗƗ**^P-values relates to the Likelihood-ratio test (omnibus test) for whether the effect of ethnicity varies with language concordance (comparing model 2 with a model where the language-ethnicity concordance effect was constrained to be constant across the three ethnic groups).

*There was no evidence (p = 0.19) to suggest that these adjusted mean scores varied across the three ethnic groups (post-hoc Wald test).

**There was evidence (p = 0.0402) to suggest that these adjusted mean scores varied across the three ethnic groups. In particular, the adjusted mean scores varied between Bangladeshi and Indian ethnic group (p = 0.0368). However, adjusted differences between Pakistani and Indian and between Pakistani and Bangladeshi ethnic groups were not significant (p = 0.10 for both) (post-hoc Wald tests).

^‡^ There was no evidence (p = 0.19) to suggest that these adjusted mean scores varied across the three ethnic groups (post-hoc Wald test).

After adjusting for age, gender, deprivation, self-rated health status, self-reported presence of a mental health condition, and a random effect for practice, we found that Bangladeshi respondents on average reported poorer communication than their White British counterparts (-2.9; 95%CI -4.2, -1.6). However, differences between practices varied according to whether a concordant language was offered or not. In practices where no concordant language was available, Bangladeshi patients reported worse experiences than White British patients (-3.3; 95%CI -4.6, -2.0). In practices where a concordant language was offered, whilst our uncertainty is large, we can say that the experience reported by Bangladeshi patients was not substantially worse than, and could have been much better than, that reported by White British patients (+4.5; 95%CI -1.0, +10.1).

As above, on average, Pakistani respondents reported poorer communication in comparison to White British respondents (-1.9; 95%CI -2.6, -1.2), but the difference in communication ratings were not substantially different to their White British counterparts when a Pakistani language was available at a practice (-0.2; 95%CI -1.5, +1.0). Pakistani respondents reported significantly worse experience in comparison to White British respondents when a Pakistani language was not offered (-2.7; 95%CI -3.6, -1.9).

Indian respondents also, on average, reported poorer experience of communication when compared to White British patients (-1.9; 95%CI -2.5, -1.4). These communication ratings were lower than their White British counterparts regardless of whether a concordant Indian language was available (*-1.5,* 95%CI -2.5, -0.5), or not available at a practice (*-2.1,* 95%CI -2.7, -1.5).

Additional files 2 and 3 details the full output of both models for single and all English practices.

### Sensitivity analysis

Repeating the analysis to include multi-doctor practices confirmed that patient reports of communication improved when a concordant language was available at a practice (see Additional file 4). The effect of language-concordance was reduced compared to the findings from single-handed practices. Nevertheless, there was significant evidence (p = 0.05) that the effect of ethnicity varies with language concordance in multi-doctor practices as well.

## Discussion

Our research has shown that Bangladeshi, Pakistani and Indian respondents reported poorer experience of doctor-patient communication than White British respondents in single-handed practices, as found in previous studies on all English practices (including single and multi-doctor practices) [20]. By linking three large national datasets, our analysis found that language and ethnicity concordance between the patient and their practice was associated with more positive reports of patient experiences and therefore may play an important role in patients’ evaluation of communication [16,17]. We found that the average difference in ratings of communication being better (for Bangladeshis) or no worse (for Pakistanis) than White British respondents in practices where a concordant language was available.

Communication scores are generally very high, even among ethnic minority patients. However, anecdotal evidence suggests that some practice may perform better than others. In order to put differences in context, we can compare communication scores to the spread seen in practice scores (i.e., one standard deviation in practice scores is equal to a difference of six points in communication scores). What we find is that the differences in scores observed are not easily dismissed when compared to the spread of average practice scores across the country, even after controlling for a number of socio-demographic variables. For example, on average, if a Bangladeshi and a White British patient were seen in the same practice, a reported difference of -3 points among Bangladeshi patients when compared to their White British counterparts is equivalent to a White British being seen in an average practice (50th percentile) and the Bangladeshi patient being seen in a practice performing at the 31st percentile. However, currently there is no sufficient understanding of the practical significance of these differences. Ongoing research is trying to gain insights into this area but further research is needed.

Although the availability of a concordant language was associated with more favourable reports of doctor-patient communication when compared to White British respondents among Pakistani and Bangladeshi respondents, this effect was smaller for Indian respondents. This might be partly explained by the difficulties in linking concordant languages to Indian respondents, as there are large language variations within the Indian category.

Nevertheless, it is important to highlight that there are substantial differences between the South Asian ethnic groups which may also explain why there was little effect for the Indian respondents. For example, those from an Indian community have been found to have higher educational qualifications than their Pakistani and Bangladeshi counterparts of working age in the UK [28,29]. There is also some indication that Indians are more likely to be proficient in English and score better on the Aberystwyth Bi-culturalism scale than Pakistanis and Bangladeshis, who more strongly emphasise their distinctive Muslim/religious identities [30]. The Aberystwyth Bi-culturalism/Acculturation scale is widely used as a tool in educational psychology [30]. It was originally devised by Ghuman [31,32] to investigate acculturation strategies between two cultures (bi-culturalism), i.e., whether South Asians in Britain preferred integration and assimilation into a more British identity, as well as other questions around the perceived level of marginalisation and separation from British culture or identity. Therefore the differences in language proficiency and lower degree of acculturation for the Pakistani and Bangladeshi groups may be mitigated when there is language (or ethnicity) concordance. Particularly, as language preference may be consciously or subconsciously affected by a person’s cultural or religious values [33], and is therefore sometimes indicative of a proxy of acculturation, rather than an ability to communicate [34].

Although we examined patient’s ethnicity matching with at least one language available at a practice, this may also indirectly infer that the ethnicity of the doctor (proxy through language) matches the patient’s ethnicity as well. There may be a number of ways in which the matching of doctors’ and patients’ language (and/or ethnicity) could affect patient reports of communication. Below, we offer four possible explanations for this effect:

1. **Better communication:** Language plays an important role in communication, therefore the availability of a language may mean doctors and patients have better communication and develop greater understanding due to the availability of a concordant language in the practice. Even when ethnic minority patients are able to speak English in their daily routine, qualitative accounts suggest that they might still find it challenging to communicate effectively due to misunderstandings related to issues of language (pronunciation, speech delivery, grammar/vocabulary) [9,35]. Using language concordant interpreters to support non-concordant doctor-patientinteractions may overcome some of these communication challenges. However, some evidence suggests that the use of interpreter services (as opposed to the doctor communicating directly with the patient) compromises aspects of communication and patient trust, for example patients using interpreters may have more questions about their health care in general, and about their mental health specifically, that they did not ask, due to the presence of a third party (interpreter) [36]. This might explain why a patient visiting a practice offering a concordant “doctor language” has strengths in overcoming such barriers, and also highlights the need for interpreters who are appropriately trained to garner patient trust in triad medical encounters. This is particularly challenging as interpretation services in English practices, when on offer, are often done over the phone rather than face-to-face [37].
2. **Cultural competency:** Cultural competency refers to the importance of reflecting and examining interpersonal relationships in health care (such as during a medical consultation) to include concerns about the patient’s well-being, show respect, and incorporate the patient’s views, personal value base and beliefs in the decision making process [17,38]. Therefore, it is possible that the availability of a South Asian language at a practice may be a marker for greater cultural competency; since patients with concordant languages available at their practice are more likely to have a doctor whose ethnicity (or cultural belief awareness) matches with them as well. Cultural competency can mean more than a doctor and a patient sharing the same ethnic group [39], however we cannot rule this out as one of the possible explanation for improvements in communication scores. Cultural competence and patient centeredness have trust at their core, which is built and maintained by an effective rapport between doctors and their patients [40]. Therefore patients may perceive or experience greater trust and engagement in clinical and health decisions with doctors who share the same ethnicity or language [15]. Cultural competency may also play a part in encouraging greater sensitivity and understanding of patients cultural and religious values, which are important dimensions of acculturation [41].
3. **Expectations or attitudes differ or discord:** A US study found that ethnicity-concordance also has an independent effect on patients’ evaluation, rather than being driven by the actual verbal nature of medical dialogue [42]. That is, even after controlling for difference in length of consultation, patient’s and doctor’s speech speed, and consultation style, the reported experience of care were more positive among patients who were seen by doctors who had the same ethnicity, as compared to those who did not share the same ethnicity category. Therefore, patient’s and doctor’s attitudes to one another may mediate their relationship during the consultation and is thereby reflected in the difference in reports of patient experience. This can be ***doctors’*** attitudes and expectations of ethnic minority patients; or ethnic minority ***patients’*** attitudes and expectations of the doctors. These expectations and attitudes are not necessarily discriminatory on either side, but rather reflect cultural variations in attitudes and expectations in what is perceived as the role of the doctor and patient during a consultation.
4. **Societal or health system discrimination:** Whilst all discrimination of patients is unacceptable and recognised as both unprofessional and unethical [2], patients’ preference of a doctor (proxy through language) whose ethnicity matches with their own self-reported ethnicity may be as a result of possible historical or personal discriminatory experiences in the health care system, or indeed society as a whole. Other studies have indicated that indirect discrimination or stereotyping of patients groups [15,43,44] may also be a reason why patients may prefer and report more positively in concordant consultations.

### Strengths and limitations

One of the strengths of this study is that it links three large national datasets to explore how the availability of a doctor who speaks a concordant South Asian language at a practice may affect reports of doctor-patient communication experience among South Asian patients. Although a practice (or doctor) offers a South Asian language, patients may not necessarily have their consultations with the doctor offering a concordant language at the practice. Single-handed practices can have other staff, such as doctors in assistant, salaried or locum capacity. However, there is some evidence to suggest that patients are more likely to see their preferred general practitioner and receive continuity of care in single-handed practices than in multi-doctor practices [45-47]. It is also possible that where Asian patients have the choice, they are more likely to overcome their linguistic and/or cultural barriers by consulting with concordant Asian doctors [48-51]. Therefore, it is more likely that in single-handed practices we would be able to detect an effect of language concordance using practice level data, such as the GP Patient Survey, if it is present, albeit with an effect size that may be diluted. As with any observational study, we cannot determine causality definitively and there may be practice factors other than the language offered (but nonetheless associated with it) which confound the association we have found. However, any effect would need to be differential across the different patient groups and no obvious candidate is known to us at this time.

Ethnicity is a complex concept and it is difficult to measure and distinguish languages between ethnic groups [52]. This is particularly so in relation to linking languages with ethnicity for Pakistani and Indian categories, where languages (e.g., Punjabi) are shared between both ethnic groups. We attempted to reduce errors due to inaccuracy of coding of the Punjabi language by examining the original NHS Choices data and found that the majority of doctors who spoke Punjabi, also spoke another Pakistani language (e.g., Urdu or Mirpuri). For that reason we assigned Punjabi to patients reporting Pakistani ethnicity only. The stronger effect size observed for Bangladeshi respondents might have also been due to the fact that there are fewer languages within this ethnic group in comparison to Indians and Pakistanis. Therefore misclassification error may be larger for Indian patients, thus attenuating the effect. In addition, the large confidence intervals for the Bangladeshi ethnic group represent the small number of respondents who were seen in a practice where a concordant Bangladeshi language was available.

There is a risk of bias resulting from the assumption that demographic variables relate to communication ratings in the same way for all ethnic groups in our regression analysis. We overcame this by carrying out a sensitivity analysis (Additional file 5), which repeated the regression (model 2) with additional interactions between ethnicity and the all of demographic variables in the model (i.e., ethnicity by gender, ethnicity by age, ethnicity by self-rated health status, ethnicity by self-reported, ethnicity by presence of a mental health condition, and ethnicity by deprivation). The test confirmed that effect of the language concordance on reported doctor-patient communication scores is the same, and that the relationships between demographic and communication is not influencing or distorting the results.

The GP Patient Survey has a response rate of 36%, which is comparable with similar national patient surveys [53,54]. Women, middle-aged patients, and those in affluent areas are more likely to respond to these surveys. A major limitation of non-response specific to this study is that patients with language problems may be excluded. For instance, an analysis of 210 published studies looking at patient experience identified language problems as a common reason for non-participation in surveys and exclusion from analyses [55]. Although the GP Patient Survey is available for Bangladeshi, Indian and Pakistani respondents in appropriate languages, the translated versions have very low uptake (i.e., 337/1,944,410 surveys were completed in a South Asian language [24]). This is further complicated, as there is no agreed written form of the main language spoken by Pakistani (Mirpuri) and Bangladeshi (Sylheti) communities in the UK [56]. This suggests that we are only getting responses from people from ethnic minorities who can speak English or possibly those who have completed the survey through a proxy (relative), so our estimates of differences are likely to be conservative.

## Conclusions

Understanding of the practical significance of communication scores differences is not sufficient and is an ongoing topic further research. However, substantial differences in reports of doctor-patient communication exist between ethnic groups [20]. Our results suggest that patient experience among Bangladeshis and Pakistanis is improved where the practice offers a language that is concordant with the patient’s ethnicity. Among Indian respondents however the average communication ratings were lower than their White British respondents, regardless of whether a concordant Indian language was available or not at a practice. This may be due to a number of socio-cultural factors (such as variations in language proficiency, educational attainment and acculturation), or other reasons unrelated to language such as differences in expectations or attitudes, and partly due to the large diversity in the languages spoken by the Indian communities living in the UK (which is possibly attenuating the effects of language concordance). This also supports previous claims that assigning Pakistanis, Bangladeshis and Indian community members to a single South Asian category may mask opportunities for exploring and improving quality of care, as there is great variation between the ethnic groups [29,57].

In light of our findings, we make the following recommendations: i) language/cultural competence should be considered when interpreting survey data; ii) increasing the cultural competence of health care practice and/or availability of languages relevant to the ethnic minority population may improve patients’ experience of care; iii) encouraging increased language support for ethnic minority patients and practices serving significant ethnic minority catchments, such as programmes to improve language skills of migrants, may improve patients’ experience of care and iv) further investigation is needed to explore the processes within a medical consultation that effect communication, including distinguishing the effect of doctors’ ethnicity from consultation language.

## Additional files

Additional file 1:
**Summary of the datasets used.**
Additional file 2:
**Differences in reports of** **doctor-patient** **communication scores, with and without the effects of language/ethnicity concordance in English practices:** **Single-handed** **practices.**
Additional file 3:
**Differences in reports of** **doctor-patient** **communication scores, with and without the effects of language/ethnicity concordance in English practices: Single &** **multi-doctor** **practices.**
Additional file 4:
**Effect of language/ethnicity concordance on the average** **doctor-patient** **communication difference for South Asians, when compared to White British respondents (sensitivity analysis in** **multi-doctor** **practices).**
Additional file 5:
**Effect of language/ethnicity concordance on the average** **doctor-patient** **communication, with and without adjusting for the relationships between demographic and communication ratings as not being the same for all ethnic groups.**
