# Supplementary material for: Does the availability of a South Asian language in practices improve reports of doctor-patient communication from South Asian patients? Cross sectional analysis of a national patient survey in English general practices
Source: BMC Fam Pract. 2015 May 6;16:55. doi: 10.1186/s12875-015-0270-5 (PMC4494805; doi:10.1186/s12875-015-0270-5)
Supplement: Additional file 1: — Summary of the datasets used. [file 12875_2015_270_MOESM1_ESM.docx]

**Additional file 1: Summary of the datasets used**

| **Dataset** | **Year** | **Source** | **Information** | **Purpose** | **How was it linked?** |
| --- | --- | --- | --- | --- | --- |
| **National GP Patient Survey (GPPS):** | 2010/2011 | **Ipsos MORI,** on behalf of NHS England (Department of Health, UK) | Patients reports of their experience in primary care | - Patients reports of doctor-patient communication - Patients’ socio-demographic information | **Main dataset** |
| **GP Census** | 2010 | **NHS Information Centre** | Numbers and details of General Practitioner in England. It includes information on their practices, staff, patients and the services they provide. | - To identify number of GP per practice (i.e. single-handed practices) | Unique practice code |
| **NHS Choice language dataset** | 2011/2012 | **NHS Choice Website,** owned by the Department of Health, UK | Includes information on practices in England, with additional languages (i.e. a language other than English) offered by each of the doctors in a practice | - To identify additional languages offered at a practice | Practice postcode (removing duplicate postcodes) |
