# Supplementary material for: Does the availability of a South Asian language in practices improve reports of doctor-patient communication from South Asian patients? Cross sectional analysis of a national patient survey in English general practices
Source: BMC Fam Pract. 2015 May 6;16:55. doi: 10.1186/s12875-015-0270-5 (PMC4494805; doi:10.1186/s12875-015-0270-5)
Supplement: Additional file 2: — Differences in reports of doctor-patient communication scores, with and without the effects of language/ethnicity concordance in English practices: Single-handed practices. [file 12875_2015_270_MOESM2_ESM.docx]

**Additional file 2: Differences in reports of doctor-patient communication scores, with and without the effects of language/ethnicity concordance in English practices: Single-handed practices**

|  |  | **Model 1** | | | **Model 2: Including concordance** | | |
| --- | --- | --- | --- | --- | --- | --- | --- |
| **Variable Category** | | **Score Difference*** | | **P-value** | **Score Difference*** | | **P-value** |
|  |  | **Difference (95% CI)** | |  | **Difference (95% CI)** | |  |
| **Gender** | |  | | **<0.0001** |  | | **<0.0001** |
|  | Male | **Reference** | |  | **Reference** | |  |
|  | Female | **-0.6** | (-0.8, -0.4) |  | **-0.6** | (-0.8, -0.4) |  |
|  |  |  |  |  |  |  |  |
| **Age** | |  |  | **<0.0001** |  |  | **<0.0001** |
|  | 18 to 24 | **-9.9** | (-10.4, -9.4) |  | **-9.9** | (-10.4, -9.3) |  |
|  | 25 to 34 | **-9.1** | (-9.5, -8.7) |  | **-9.1** | (-9.5, -8.7) |  |
|  | 35 to 44 | **-4.8** | (-5.1, -4.4) |  | **-4.8** | (-5.1, -4.4) |  |
|  | 45 to 54 | **-2.3** | (-2.7, -2.0) |  | **-2.3** | (-2.7, -2.0) |  |
|  | 55 to 64 | **Reference** | |  | **Reference** | |  |
|  | 65 to 74 | **3.1** | (2.7, 3.4) |  | **3.1** | (2.7, 3.4) |  |
|  | 75 to 84 | **4.1** | (3.7, 4.5) |  | **4.1** | (3.7, 4.5) |  |
|  | 85+ | **3.3** | (2.6, 4.0) |  | **3.3** | (2.6, 4.0) |  |
|  |  |  |  |  |  |  |  |
| **Ethnicity** | |  |  | **<0.0001** |  |  | **<0.0001** |
| ***White*** | White British | **Reference** | |  | **Reference** | |  |
|  | Irish | **0.6** | (-0.2, 1.4) |  | **0.6** | (-0.2, 1.4) |  |
|  | Any other White background | **-3.4** | (-3.8, -2.9) |  | **-3.4** | (-3.8, -2.9) |  |
| ***Mixed*** | White and Black Caribbean | **-0.3** | (-2.3, 1.6) |  | **-0.3** | (-2.3, 1.6) |  |
|  | White and Black African | **1.2** | (-1.0, 3.4) |  | **1.2** | (-1.0, 3.4) |  |
|  | White and Asian | **-2.6** | (-4.6, -0.6) |  | **-2.6** | (-4.6, -0.6) |  |
|  | Any other Mixed background | **-2.7** | (-4.4, -1.0) |  | **-2.7** | (-4.4, -1.0) |  |
| ***South Asian*** | Indian | **-1.9** | (-2.5, -1.4) |  | **-2.1** | (-2.7, -1.5) |  |
|  | Pakistani | **-1.9** | (-2.6, -1.2) |  | **-2.7** | (-3.6, -1.9) |  |
|  | Bangladeshi | **-2.9** | (-4.2, -1.6) |  | **-3.3** | (-4.6, -2.0) |  |
|  | Any other Asian background | **-1.2** | (-1.9, -0.6) |  | **-1.2** | (-1.9, -0.5) |  |
| ***Black*** | Black Caribbean | **-0.4** | (-1.2, 0.4) |  | **-0.4** | (-1.2, 0.4) |  |
|  | Black African | **0.6** | (-0.1, 1.3) |  | **0.6** | (-0.1, 1.3) |  |
|  | Any other Black background | **1.1** | (-0.2, 2.3) |  | **1.1** | (-0.2, 2.3) |  |
| ***Chinese*** | Chinese | **-5.1** | (-6.4, -3.9) |  | **-5.1** | (-6.4, -3.9) |  |
| ***Other ethnic group*** | Any other ethnic group | **-2.1** | (-2.7, -1.6) |  | **-2.1** | (-2.7, -1.6) |  |
|  |  |  |  |  |  |  |  |
| **Deprivation** | |  |  | **0.4394** |  |  | **0.4419** |
|  | "1" (least deprived) | **Reference** | |  | **Reference** | |  |
|  | "2" | **-0.3** | (-0.7, 0.2) |  | **-0.3** | (-0.7, 0.2) |  |
|  | "3" | **-0.4** | (-0.9, 0.1) |  | **-0.4** | (-0.9, 0.1) |  |
|  | "4" | **-0.3** | (-0.8, 0.2) |  | **-0.3** | (-0.8, 0.2) |  |
|  | "5" (most deprived) | **-0.2** | (-0.6, 0.3) |  | **-0.2** | (-0.6, 0.3) |  |
|  |  |  |  |  |  |  |  |
| **Self-reported health status** | |  |  | **<0.0001** |  |  | **<0.0001** |
|  | Excellent | **Reference** | |  | **Reference** | |  |
|  | Very good | **-4.2** | (-4.6, -3.8) |  | **-4.2** | (-4.6, -3.8) |  |
|  | Good | **-8.0** | (-8.4, -7.6) |  | **-8.0** | (-8.4, -7.6) |  |
|  | Fair | **-10.0** | (-10.5, -9.6) |  | **-10.9** | (-10.5, -9.6) |  |
|  | Poor | **-10.8** | (-11.3, -10.3) |  | **-10.8** | (-11.3, -10.3) |  |
|  |  |  |  |  |  |  |  |
| **Long-standing psychological or emotional condition** | |  |  | **0.0102** |  |  | **0.0101** |
|  | No | **Reference** | |  | **Reference** | |  |
|  | Yes | **0.6** | (0.1, 1.0) |  | **0.6** | (0.1, 1.0) |  |
|  |  |  |  |  |  |  |  |
| **Language-ethnicity concordance:** | |  |  |  |  |  | **0.0004** |
|  |  |  |  |  |  |  |  |
| ***Bangladeshi*** |  |  |  |  |  |  |  |
|  | **No** | **n/a** | |  | **Reference** | |  |
|  | **Yes** |  |  |  | **7.8** | (2.1, 13.6) |  |
| ***Indian*** |  |  |  |  |  |  |  |
|  | **No** | **n/a** | |  | **Reference** | |  |
|  | **Yes** |  |  |  | **0.6** | (-0.5, 1.8) |  |
| ***Pakistani*** |  |  |  |  |  |  |  |
|  | **No** | **n/a** | |  | **Reference** | |  |
|  | **Yes** |  |  |  | **2.5** | (1.0, 4.0) |  |
| *** Coefficients were also adjusted for a random effect for practice**  **Score difference (scale 0-100) in reports of doctor patient communication** | | | | | | | |
