# Supplementary material for: Does the availability of a South Asian language in practices improve reports of doctor-patient communication from South Asian patients? Cross sectional analysis of a national patient survey in English general practices
Source: BMC Fam Pract. 2015 May 6;16:55. doi: 10.1186/s12875-015-0270-5 (PMC4494805; doi:10.1186/s12875-015-0270-5)
Supplement: Additional file 3: — Differences in reports of doctor-patient communication scores, with and without the effects of language/ethnicity concordance in English practices: Single & multi-doctor practices. [file 12875_2015_270_MOESM3_ESM.docx]

**Additional file 3: Differences in reports of doctor-patient communication scores, with and without the effects of language/ethnicity concordance in English practices: Single & multi-doctor practices**

|  |  | **Model 1** | | | **Model 2: Including concordance** | | |
| --- | --- | --- | --- | --- | --- | --- | --- |
| **Variable Category** | | **Score Difference*** | | **P-value** | **Score Difference*** | | **P-value** |
|  |  | **Difference (95% CI)** | |  | **Difference (95% CI)** | |  |
| **Gender** | |  | | **0.9240** |  | | **0.9179** |
|  | Male | **Reference** | |  | **Reference** | |  |
|  | Female | **0.0** | (-0.1, 0.1) |  | **0.0** | (-0.1, 0.1) |  |
|  |  |  |  |  |  |  |  |
| **Age** | |  |  | **<0.0001** |  |  | **<0.0001** |
|  | 18 to 24 | **-8.6** | (-8.8, -8.4) |  | **-8.6** | (-8.8, -8.4) |  |
|  | 25 to 34 | **-7.8** | (-7.9, -7.7) |  | **-7.8** | (-7.9, -7.7) |  |
|  | 35 to 44 | **-4.7** | (-4.8, -4.6) |  | **-4.7** | (-4.8, -4.6) |  |
|  | 45 to 54 | **-2.6** | (-2.7, -2.5) |  | **-2.6** | (-2.7, -2.5) |  |
|  | 55 to 64 | **Reference** | |  | **Reference** | |  |
|  | 65 to 74 | **2.9** | (2.8, 3.0) |  | **2.9** | (2.8, 3.0) |  |
|  | 75 to 84 | **4.0** | (3.9, 4.1) |  | **4.0** | (3.9, 4.1) |  |
|  | 85+ | **3.6** | (3.4, 3.8) |  | **3.6** | (3.4, 3.8) |  |
|  |  |  |  |  |  |  |  |
| **Ethnicity** | |  |  | **<0.0001** |  |  | **<0.0001** |
| ***White*** | White British | **Reference** | |  | **Reference** | |  |
|  | Irish | **0.6** | (0.3, 0.9) |  | **0.6** | (0.3, 0.9) |  |
|  | Any other White background | **-3.5** | (-3.7, -3.4) |  | **-3.5** | (-3.7, -3.4) |  |
| ***Mixed*** | White and Black Caribbean | **-0.4** | (-1.1, 0.3) |  | **-0.4** | (-1.1, 0.3) |  |
|  | White and Black African | **-1.5** | (-2.4, -0.6) |  | **-1.5** | (-2.4, -0.6) |  |
|  | White and Asian | **-2.5** | (-3.2, -1.8) |  | **-2.5** | (-3.2, -1.8) |  |
|  | Any other Mixed background | **-2.6** | (-3.2, -2.0) |  | **-2.6** | (-3.2, -2.0) |  |
| ***South Asian*** | Indian | **-2.5** | (-2.9, -2.4) |  | **-2.7** | (-2.9, -2.4) |  |
|  | Pakistani | **-3.1** | (-3.7, -3.0) |  | **-3.3** | (-3.7, -3.0) |  |
|  | Bangladeshi | **-3.5** | (-4.3, -3.2) |  | **-3.7** | (-4.3, -3.2) |  |
|  | Any other Asian background | **-1.8** | (-2.1, -1.5) |  | **-1.8** | (-2.1, -1.5) |  |
| ***Black*** | Black Caribbean | **-0.3** | (-0.6, 0.0) |  | **-0.3** | (-0.6, 0.0) |  |
|  | Black African | **0.4** | (0.2, 0.7) |  | **0.4** | (0.2, 0.7) |  |
|  | Any other Black background | **0.5** | (0.0, 1.0) |  | **0.5** | (0.0, 1.0) |  |
| ***Chinese*** | Chinese | **-5.9** | (-6.4, -5.5) |  | **-5.9** | (-6.4, -5.5) |  |
| ***Other ethnic group*** | Any other ethnic group | **-2.3** | (-2.5, -2.1) |  | **-2.3** | (-2.5, -2.1) |  |
|  |  |  |  |  |  |  |  |
| **Deprivation** | |  |  | **<0.0001** |  |  | **<0.0001** |
|  |  |  |  |  |  |  |  |
|  | "1" (least deprived) | **Reference** | |  | **Reference** | |  |
|  | "2" | **0.1** | (0.0, 0.2) |  | **0.1** | (0.0, 0.2) |  |
|  | "3" | **0.2** | (0.0, 0.3) |  | **0.2** | (0.0, 0.3) |  |
|  | "4" | **0.2** | (0.1, 0.4) |  | **0.2** | (0.1, 0.4) |  |
|  | "5" (most deprived) | **0.7** | (0.5, 0.8) |  | **0.7** | (0.5, 0.8) |  |
|  |  |  |  |  |  |  |  |
| **Self-reported health status** | |  |  | **<0.0001** |  |  | **<0.0001** |
|  | Excellent | **Reference** | |  | **Reference** | |  |
|  | Very good | **-3.3** | (-3.5, -3.2) |  | **-3.3** | (-3.5, -3.2) |  |
|  | Good | **-6.5** | (-6.6, -6.4) |  | **-6.5** | (-6.6, -6.4) |  |
|  | Fair | **-8.0** | (-8.1, -7.8) |  | **-8.0** | (-8.1, -7.8) |  |
|  | Poor | **-8.2** | (-8.4, -8.1) |  | **-8.2** | (-8.4, -8.1) |  |
|  |  |  |  |  |  |  |  |
| **Long-standing psychological or emotional condition** | |  |  | **<0.0001** |  |  | **<0.0001** |
|  | No | **Reference** | |  | **Reference** | |  |
|  | Yes | **1.7** | (1.6, 1.9) |  | **1.7** | (1.6, 1.9) |  |
|  |  |  |  |  |  |  |  |
| **Language-ethnicity concordance:** | |  |  |  |  |  | **<0.0001** |
|  |  |  |  |  |  |  |  |
| ***Bangladeshi*** |  |  |  |  |  |  |  |
|  | **No** | **n/a** | |  | **Reference** | |  |
|  | **Yes** |  |  |  | **3.3** | (1.4, 5.3) |  |
| ***Indian*** |  |  |  |  |  |  |  |
|  | **No** | **n/a** | |  | **Reference** | |  |
|  | **Yes** |  |  |  | **0.8** | (0.3, 1.4) |  |
| ***Pakistani*** |  |  |  |  |  |  |  |
|  | **No** | **n/a** | |  | **Reference** | |  |
|  | **Yes** |  |  |  | **0.9** | (0.2, 1.6) |  |
| *** Coefficients were also adjusted for a random effect for practice**  **Score difference (scale 0-100) in reports of doctor patient communication** | | | | | | | |
