# Supplementary material for: Does the availability of a South Asian language in practices improve reports of doctor-patient communication from South Asian patients? Cross sectional analysis of a national patient survey in English general practices
Source: BMC Fam Pract. 2015 May 6;16:55. doi: 10.1186/s12875-015-0270-5 (PMC4494805; doi:10.1186/s12875-015-0270-5)
Supplement: Additional file 4: — Effect of language/ethnicity concordance on the average doctor-patient communication difference for South Asians, when compared to White British respondents (sensitivity analysis in multi-doctor practices). [file 12875_2015_270_MOESM4_ESM.docx]

**Additional file 4: Effect of language/ethnicity concordance on the average doctor-patient communication difference for South Asians, when compared to White British respondents (sensitivity analysis in multi-doctor practices)**

| **Ethnic Group** | **Model 1:** | **Model 2:** | |  |
| --- | --- | --- | --- | --- |
|  | **Average difference compared to White British respondents** | **Average difference compared to White British respondents when a concordant language is:** | | |
|  |  | ***Available at practice*** | ***Not available at practice*** | |
| Indian | -2.5 (-2.8, -2.3) | -1.8 (-2.4, -1.3) | -2.7 (-2.9, -2.4) | |
| Pakistani | -3.1 (-3.4, -2.8) | -2.4 (-3.0, -1.8) | -3.3 (-3.7, -3.0) | |
| Bangladeshi | -3.5 (-4.0, -3.0) | -0.4 (-2.3, 1.5) | -3.7 (-4.3, -3.2) | |
|  | **p<0.0001 Ɨ** | **Likelihood-ratio test: p=0.0501 ƗƗ** | | |
| Both models were adjusted for age, gender, deprivation, self-rated health status, presence of a mental health condition, and a random effect for practice | | | | |

**Ɨ** Joint test of the differences of South Asians from White British

**ƗƗ** P-values relates to the Likelihood-ratio test (omnibus test) for whether the effect of ethnicity varies with language concordance
