# Supplementary material for: Does the availability of a South Asian language in practices improve reports of doctor-patient communication from South Asian patients? Cross sectional analysis of a national patient survey in English general practices
Source: BMC Fam Pract. 2015 May 6;16:55. doi: 10.1186/s12875-015-0270-5 (PMC4494805; doi:10.1186/s12875-015-0270-5)
Supplement: Additional file 5: — Effect of language/ethnicity concordance on the average doctor-patient communication, with and without adjusting for the relationships between demographic and communication ratings as not being the same for all ethnic groups. [file 12875_2015_270_MOESM5_ESM.docx]

**Additional file 5: Effect of language/ethnicity concordance on the average doctor-patient communication, with and without adjusting for the relationships between demographic and communication ratings as not being the same for all ethnic groups**

| ***Language-ethnicity concordance:*** | **Main results** | | **Including ethnicity interactions (sensitivity analysis)*** | |
| --- | --- | --- | --- | --- |
|  | *Average difference*  *Score (95%CI)* | *P-value* | *Average difference*  *Score (95%CI)* | *P-value* |
| *Bangladeshi* | 7.8 (2.1, 13.6) | 0.007 | 7.4 (1.6, 13.2) | 0.013 |
| *Indian* | 0.6 (-0.5, 1.8) | 0.285 | 0.6 (-0.5, 1.8) | 0.296 |
| *Pakistani* | 2.5 (1.0, 4.0) | 0.001 | 2.5 (1.0, 4.0) | 0.001 |

***** Adjusting for the relationships between demographic and communication ratings as not being the same for all ethnic groups: Regression (model 2) repeated with additional interactions between ethnicity and the all of demographic variables in the model (i.e., ethnicity by gender, ethnicity by age, ethnicity by self-rated health status, ethnicity by self-reported, ethnicity by presence of a mental health condition, and ethnicity by deprivation).
